# Supplementary material for: Pyruvate Dehydrogenase Kinase 1 inhibition mediated oxidative phosphorylation enhancement in cartilage promotes osteoarthritis progression
Source: BMC Musculoskelet Disord. 2023 Jul 20;24:597. doi: 10.1186/s12891-023-06585-6 (PMC10357736; doi:10.1186/s12891-023-06585-6)
Supplement: Supplementary file 1 — Additional file 1. [file 12891_2023_6585_MOESM1_ESM.zip › KEGG.Gsea.1653622432464/KEGG_NOD_LIKE_RECEPTOR_SIGNALING_PATHWAY.html]

Details for gene set KEGG\_NOD\_LIKE\_RECEPTOR\_SIGNALING\_PATHWAY[GSEA]

|  || Dataset | 55457\_collapsed\_to\_symbols.cls.cls #OA\_versus\_normal.cls.cls #OA\_versus\_normal\_repos |
| Phenotype | cls.cls#OA\_versus\_normal\_repos |
| Upregulated in class | normal |
| GeneSet | KEGG\_NOD\_LIKE\_RECEPTOR\_SIGNALING\_PATHWAY |
| Enrichment Score (ES) | -0.3404555 |
| Normalized Enrichment Score (NES) | -1.3229434 |
| Nominal p-value | 0.08153078 |
| FDR q-value | 0.25647774 |
| FWER p-Value | 0.999 |
Table: GSEA Results Summary

  

Fig 1: Enrichment plot: KEGG\_NOD\_LIKE\_RECEPTOR\_SIGNALING\_PATHWAY      
 Profile of the Running ES Score & Positions of GeneSet Members on the Rank Ordered List

  

| SYMBOL | TITLE | RANK IN GENE LIST | RANK METRIC SCORE | RUNNING ES | CORE ENRICHMENT || 1 | NLRP1 | NLR family pyrin domain containing 1 [Source:HGNC Symbol;Acc:HGNC:14374] | 200 | 0.819 | 0.0227 | No |
| 2 | CASP1 | caspase 1 [Source:HGNC Symbol;Acc:HGNC:1499] | 431 | 0.694 | 0.0371 | No |
| 3 | TRIP6 | thyroid hormone receptor interactor 6 [Source:HGNC Symbol;Acc:HGNC:12311] | 524 | 0.650 | 0.0603 | No |
| 4 | MAPK9 | mitogen-activated protein kinase 9 [Source:HGNC Symbol;Acc:HGNC:6886] | 721 | 0.588 | 0.0725 | No |
| 5 | MAPK3 | mitogen-activated protein kinase 3 [Source:HGNC Symbol;Acc:HGNC:6877] | 757 | 0.579 | 0.0968 | No |
| 6 | TAB2 | TGF-beta activated kinase 1 (MAP3K7) binding protein 2 [Source:HGNC Symbol;Acc:HGNC:17075] | 877 | 0.549 | 0.1132 | No |
| 7 | PYCARD | PYD and CARD domain containing [Source:HGNC Symbol;Acc:HGNC:16608] | 1164 | 0.484 | 0.1135 | No |
| 8 | MAP3K7 | mitogen-activated protein kinase kinase kinase 7 [Source:HGNC Symbol;Acc:HGNC:6859] | 1701 | 0.399 | 0.0904 | No |
| 9 | MEFV | "MEFV innate immuity regulator, pyrin [Source:HGNC Symbol;Acc:HGNC:6998]" | 2020 | 0.359 | 0.0824 | No |
| 10 | TNF | tumor necrosis factor [Source:HGNC Symbol;Acc:HGNC:11892] | 2098 | 0.350 | 0.0928 | No |
| 11 | MAPK14 | mitogen-activated protein kinase 14 [Source:HGNC Symbol;Acc:HGNC:6876] | 2352 | 0.320 | 0.0880 | No |
| 12 | IL18 | interleukin 18 [Source:HGNC Symbol;Acc:HGNC:5986] | 2472 | 0.306 | 0.0930 | No |
| 13 | MAPK13 | mitogen-activated protein kinase 13 [Source:HGNC Symbol;Acc:HGNC:6875] | 2544 | 0.298 | 0.1014 | No |
| 14 | NOD2 | nucleotide binding oligomerization domain containing 2 [Source:HGNC Symbol;Acc:HGNC:5331] | 3154 | 0.235 | 0.0650 | No |
| 15 | CASP5 | caspase 5 [Source:HGNC Symbol;Acc:HGNC:1506] | 3302 | 0.221 | 0.0638 | No |
| 16 | CCL13 | C-C motif chemokine ligand 13 [Source:HGNC Symbol;Acc:HGNC:10611] | 3379 | 0.214 | 0.0679 | No |
| 17 | RELA | "RELA proto-oncogene, NF-kB subunit [Source:HGNC Symbol;Acc:HGNC:9955]" | 3942 | 0.161 | 0.0317 | No |
| 18 | NFKB1 | nuclear factor kappa B subunit 1 [Source:HGNC Symbol;Acc:HGNC:7794] | 4626 | 0.102 | -0.0168 | No |
| 19 | RIPK2 | receptor interacting serine/threonine kinase 2 [Source:HGNC Symbol;Acc:HGNC:10020] | 4848 | 0.083 | -0.0301 | No |
| 20 | BIRC2 | baculoviral IAP repeat containing 2 [Source:HGNC Symbol;Acc:HGNC:590] | 5054 | 0.069 | -0.0428 | No |
| 21 | PSTPIP1 | proline-serine-threonine phosphatase interacting protein 1 [Source:HGNC Symbol;Acc:HGNC:9580] | 5603 | 0.024 | -0.0844 | No |
| 22 | TAB1 | TGF-beta activated kinase 1 (MAP3K7) binding protein 1 [Source:HGNC Symbol;Acc:HGNC:18157] | 5684 | 0.017 | -0.0898 | No |
| 23 | TRAF6 | TNF receptor associated factor 6 [Source:HGNC Symbol;Acc:HGNC:12036] | 5761 | 0.011 | -0.0952 | No |
| 24 | IKBKB | inhibitor of nuclear factor kappa B kinase subunit beta [Source:HGNC Symbol;Acc:HGNC:5960] | 5914 | 0.001 | -0.1070 | No |
| 25 | BIRC3 | baculoviral IAP repeat containing 3 [Source:HGNC Symbol;Acc:HGNC:591] | 5942 | -0.001 | -0.1090 | No |
| 26 | CCL5 | C-C motif chemokine ligand 5 [Source:HGNC Symbol;Acc:HGNC:10632] | 6584 | -0.048 | -0.1567 | No |
| 27 | CARD9 | caspase recruitment domain family member 9 [Source:HGNC Symbol;Acc:HGNC:16391] | 6645 | -0.053 | -0.1589 | No |
| 28 | MAPK1 | mitogen-activated protein kinase 1 [Source:HGNC Symbol;Acc:HGNC:6871] | 6853 | -0.068 | -0.1719 | No |
| 29 | CCL11 | C-C motif chemokine ligand 11 [Source:HGNC Symbol;Acc:HGNC:10610] | 6907 | -0.073 | -0.1726 | No |
| 30 | NLRP3 | NLR family pyrin domain containing 3 [Source:HGNC Symbol;Acc:HGNC:16400] | 8014 | -0.156 | -0.2514 | No |
| 31 | MAPK12 | mitogen-activated protein kinase 12 [Source:HGNC Symbol;Acc:HGNC:6874] | 8161 | -0.168 | -0.2549 | No |
| 32 | CCL7 | C-C motif chemokine ligand 7 [Source:HGNC Symbol;Acc:HGNC:10634] | 8351 | -0.182 | -0.2611 | No |
| 33 | XIAP | X-linked inhibitor of apoptosis [Source:HGNC Symbol;Acc:HGNC:592] | 8750 | -0.212 | -0.2822 | No |
| 34 | MAPK8 | mitogen-activated protein kinase 8 [Source:HGNC Symbol;Acc:HGNC:6881] | 8956 | -0.228 | -0.2875 | No |
| 35 | HSP90B1 | heat shock protein 90 beta family member 1 [Source:HGNC Symbol;Acc:HGNC:12028] | 9134 | -0.242 | -0.2900 | No |
| 36 | CXCL1 | C-X-C motif chemokine ligand 1 [Source:HGNC Symbol;Acc:HGNC:4602] | 9528 | -0.276 | -0.3077 | No |
| 37 | MAPK10 | mitogen-activated protein kinase 10 [Source:HGNC Symbol;Acc:HGNC:6872] | 9699 | -0.291 | -0.3073 | No |
| 38 | CCL2 | C-C motif chemokine ligand 2 [Source:HGNC Symbol;Acc:HGNC:10618] | 9819 | -0.303 | -0.3024 | No |
| 39 | HSP90AB1 | heat shock protein 90 alpha family class B member 1 [Source:HGNC Symbol;Acc:HGNC:5258] | 10060 | -0.327 | -0.3058 | No |
| 40 | CASP8 | caspase 8 [Source:HGNC Symbol;Acc:HGNC:1509] | 10442 | -0.366 | -0.3184 | No |
| 41 | CCL8 | C-C motif chemokine ligand 8 [Source:HGNC Symbol;Acc:HGNC:10635] | 10529 | -0.376 | -0.3075 | No |
| 42 | NOD1 | nucleotide binding oligomerization domain containing 1 [Source:HGNC Symbol;Acc:HGNC:16390] | 10953 | -0.424 | -0.3206 | Yes |
| 43 | IKBKG | inhibitor of nuclear factor kappa B kinase regulatory subunit gamma [Source:HGNC Symbol;Acc:HGNC:5961] | 10986 | -0.428 | -0.3031 | Yes |
| 44 | NFKBIB | NFKB inhibitor beta [Source:HGNC Symbol;Acc:HGNC:7798] | 11277 | -0.468 | -0.3039 | Yes |
| 45 | MAPK11 | mitogen-activated protein kinase 11 [Source:HGNC Symbol;Acc:HGNC:6873] | 11501 | -0.501 | -0.2978 | Yes |
| 46 | CHUK | component of inhibitor of nuclear factor kappa B kinase complex [Source:HGNC Symbol;Acc:HGNC:1974] | 11624 | -0.521 | -0.2830 | Yes |
| 47 | IL1B | interleukin 1 beta [Source:HGNC Symbol;Acc:HGNC:5992] | 12105 | -0.622 | -0.2914 | Yes |
| 48 | NAIP | NLR family apoptosis inhibitory protein [Source:HGNC Symbol;Acc:HGNC:7634] | 12174 | -0.637 | -0.2669 | Yes |
| 49 | CXCL8 | C-X-C motif chemokine ligand 8 [Source:HGNC Symbol;Acc:HGNC:6025] | 12297 | -0.676 | -0.2449 | Yes |
| 50 | IL6 | interleukin 6 [Source:HGNC Symbol;Acc:HGNC:6018] | 12460 | -0.730 | -0.2234 | Yes |
| 51 | ERBIN | erbb2 interacting protein [Source:HGNC Symbol;Acc:HGNC:15842] | 12535 | -0.765 | -0.1934 | Yes |
| 52 | TNFAIP3 | TNF alpha induced protein 3 [Source:HGNC Symbol;Acc:HGNC:11896] | 12631 | -0.814 | -0.1628 | Yes |
| 53 | HSP90AA1 | heat shock protein 90 alpha family class A member 1 [Source:HGNC Symbol;Acc:HGNC:5253] | 12730 | -0.893 | -0.1288 | Yes |
| 54 | CXCL2 | C-X-C motif chemokine ligand 2 [Source:HGNC Symbol;Acc:HGNC:4603] | 12733 | -0.896 | -0.0871 | Yes |
| 55 | NFKBIA | NFKB inhibitor alpha [Source:HGNC Symbol;Acc:HGNC:7797] | 12817 | -1.001 | -0.0468 | Yes |
| 56 | CARD8 | caspase recruitment domain family member 8 [Source:HGNC Symbol;Acc:HGNC:17057] | 12873 | -1.139 | 0.0021 | Yes |
Table: GSEA details [plain text format]

  

Fig 2: KEGG\_NOD\_LIKE\_RECEPTOR\_SIGNALING\_PATHWAY      
 Blue-Pink O' Gram in the Space of the Analyzed GeneSet

  

Fig 3: KEGG\_NOD\_LIKE\_RECEPTOR\_SIGNALING\_PATHWAY: Random ES distribution      
 Gene set null distribution of ES for **KEGG\_NOD\_LIKE\_RECEPTOR\_SIGNALING\_PATHWAY**
